# Evaluating the Impact of Urolithin A Supplementation on Running Performance, Recovery, and Mitochondrial Biomarkers in Highly Trained Male Distance Runners

**DOI:** 10.1007/s40279-025-02292-5

**Published:** 2025-08-21

**Authors:** Jamie Whitfield, Alannah K. A. McKay, Nicolin Tee, Rachel McCormick, Aimee Morabito, Leonidas G. Karagounis, Andréane M. Fouassier, Davide D’Amico, Anurag Singh, Louise M. Burke, John A. Hawley

**Affiliations:** 1 Mary MacKillop Institute for Health Research, Australian Catholic University, Melbourne, VIC Australia; 2 Institute of Social and Preventive Medicine, University of Bern, Bern, Switzerland; 3 Nestlé Health Science, Avenue Nestlé 55, 1800 Vevey, Switzerland; 4 Amazentis SA, EPFL Innovation Park, Bâtiment C, 1015 Lausanne, Switzerland; 5 Department of Sport and Exercise Sciences, Manchester Metropolitan University Institute of Sport, Manchester, UK

## Abstract

**Background:**

Urolithin A (UA) is a metabolite produced by gut bacteria following the consumption of ellagitannin-rich foods. Clinical trials in middle-aged and older adults demonstrated that supplementation with UA improves muscle strength, endurance, and biomarkers of mitochondrial health, suggesting that UA may be an effective ergogenic aid in other populations.

**Methods:**

In this double-blind, parallel group, placebo-controlled clinical trial (NCT04783207), competitive male distance runners (*n* = 42, 27.2 ± 1.0 years, $\dot{V}{\text{O}}_{{{\text{2max}}}}$ 66.4 ± 0.6 mL·kg^−1^·min^−1^, mean ± SEM) were randomized to consume either 1000 mg·day^−1^ UA (*n* = 22) or placebo (PL; *n* = 20) for 4 weeks during an altitude training camp (~ 1700–2200 m). Physiological outcomes including body composition, hemoglobin mass, running economy, and maximal aerobic capacity ($\dot{V}{\text{O}}_{{{\text{2max}}}}$) were measured in all subjects at baseline and at the end of the 4-week camp to assess training- and supplementation-induced adaptations. During the camp, a weekly downhill running bout was performed to challenge skeletal muscle, with capillary blood samples collected to assess inflammation (C-reactive protein; CRP) and indirect markers of muscle damage (creatine kinase; CK). A subset of athletes also either completed a 3000 m track time trial (*n* = 11 PL, *n* = 11 UA) or had skeletal muscle biopsies taken (*n* = 9 PL, *n* = 11 UA) pre/post supplementation to determine the effect of UA on running performance and for exploration of alterations in skeletal muscle proteome and mitochondrial function, respectively.

**Results:**

Running performance (3000 m time trial) was not significantly improved in either treatment group (UA; *p* = 0.116, PL; *p* = 0.771), although UA supplementation significantly lowered ratings of perceived exertion (RPE, *p* = 0.02) and reduced indirect markers of post-exercise muscle damage (CK, total area under the curve *p* < 0.0001) following the 3000 m time trial compared with PL. Although there was no statistically significant time × treatment interaction for aerobic capacity (*p* = 0.138), UA supplementation showed a large within-group increase in $\dot{V}{\text{O}}_{{{\text{2max}}}}$ (5.4 ± 0.9%, 66.4 ± 0.8 to 70.0 ± 1.0 mL·kg^−1^·min^−1^, *p* = 0.009, *d* = − 0.83), with a smaller increase in the PL group (3.6 ± 1.3%, 66.4 ± 0.9 to 68.7 ± 1.0 mL·kg^−1^·min^−1^, *p* = 0.098, *d* = − 0.54). Proteomic screening of skeletal muscle biopsies revealed UA upregulated pathways associated with mitochondria, while downregulating inflammatory pathways. While not statistically significant, UA led to a medium effect for increased markers of mitophagy (*d* = − 0.74), without changes in mitochondrial function.

**Conclusions:**

Our results show that 4 weeks of daily UA supplementation facilitates recovery by downregulating inflammatory pathways and indirect markers of muscle damage. However, despite a reduction in rating of exertion and increased aerobic capacity, UA supplementation did not further enhance performance in highly trained male endurance athletes.

**Supplementary Information:**

The online version contains supplementary material available at 10.1007/s40279-025-02292-5.

## Key Points

**Table:** 

Clinical trials in both middle-aged and elderly humans demonstrate that supplementation with urolithin A (UA) improves biomarkers of health including resistance to fatigue, muscle strength, and maximal aerobic capacity ($\dot{V}{\text{O}}_{{{\text{2max}}}}$) and reduces inflammation, suggesting UA may be an effective ergogenic aid to improve recovery and performance in athletes.
Highly trained male distance runners were separated into two groups supplementing with either 1000 mg·day^−1^ UA or placebo (PL) for 4 weeks while undergoing an altitude training camp at 1700–2200 m. Testing was performed at baseline and following the training camp to determine training- and supplement-induced effects on skeletal muscle function, whole-body adaptations, and race performance.
UA supplementation significantly increased $\dot{V}{\text{O}}_{{{\text{2max}}}}$ and reduced ratings of perceived exertion and indirect markers of muscle damage following exercise. However, despite the increase in aerobic capacity, there was no change in 3000 m time trial performance.

## Introduction

The regulation of mitochondria is a key component of metabolic health and disease states, with mitochondrial “dysfunction” associated with a host of metabolic disorders. As such, there is interest in interventions that can promote the creation of new mitochondria (mitochondrial biogenesis), or improve the regulation of mitochondrial quality control through mitochondrial fission, fusion, and/or autophagy (mitophagy) to improve health [1]. Urolithin A (UA) is a naturally occurring postbiotic metabolite derived via gut microflora conversion of ellagitannin and ellagic acid that stimulates both mitophagy and mitochondrial biogenesis, thereby restoring respiratory capacity in cell models and the nematode *Caenorhabditis elegans* [2]. Clinical trials in both middle-aged and elderly human participants demonstrate that supplementation with purified UA improves biomarkers of health [3, 4]. Specifically, daily supplementation with 1000 mg UA for 4 months improved muscle resistance to fatigue, muscle strength, and maximal aerobic capacity ($\dot{V}{\text{O}}_{{{\text{2max}}}}$) by ~ 10%, leading to clinically meaningful improvements in a 6-min walk, a proxy for whole-body function and performance [3, 4]. Analysis of skeletal muscle biopsy samples from several cohorts demonstrated that UA consistently upregulated mitochondrial gene [5] and protein [4] expression. Levels of plasma acylcarnitines [4, 5] and C-reactive protein (CRP) were also reduced [4], suggesting UA exerts an anti-inflammatory effect. Collectively, these studies [3–5] demonstrate that UA ingestion improves skeletal muscle work capacity through an overall increase in mitochondrial quality control and function, even in the absence of an exercise training intervention.

While the role of mitophagy in response to acute and chronic exercise in human skeletal muscle is currently unclear [6], increases in mitochondrial biogenesis and aerobic capacity are well-established phenotypic adaptations to exercise training [7]. Altitude training is a traditional and widely used approach to improve aerobic capacity and performance in endurance athletes [8]. Several training paradigms exist that manipulate the duration and degree of hypoxic exposure and subsequent adaptations (reviewed in ref. [8]). Regardless of the method employed, the primary cited mechanism for enhanced performance following altitude exposure is augmented hypoxia-inducible factor 1 (HIF-1) signaling resulting in an increased erythropoietic response [9]. However, non-hematological adaptations beneficial for athletic performance have also been reported [10]. Indeed, HIF-1 is a potent activator of wide host of gene targets containing hypoxia response elements, including those associated with angiogenesis [11], glycolysis [12], as well as substrate transport and pH regulation [13]. Some studies have also demonstrated an improvement in running economy (i.e., lower oxygen cost for a given velocity) [14], although this finding has not been universal [15]. However, prolonged altitude exposure typically induces inflammatory and redox stress, thereby challenging the athletes’ ability to recover [16, 17].

Given that skeletal muscle respiratory capacity and $\dot{V}{\text{O}}_{{{\text{2max}}}}$ are key determinants of endurance performance [18–20], targeting improvements in mitochondrial function through UA supplementation in combination with a potent altitude-induced training stimulus may be a novel strategy for athletes to improve performance outcomes, as the anti-inflammatory properties of UA may help facilitate post-exercise recovery for athletes training in these environmental conditions. Therefore, in the current study, we tested the hypothesis that supplementation with 1000 mg·day^−1^ UA for 4 weeks would enhance recovery through decreases in inflammation and indirect markers of muscle damage while improving mitochondrial function. Such adaptations would be expected to enhance endurance running performance compared to athletes consuming a placebo (PL) and undertaking the same training regimen during an intensified training camp at low to moderate altitude.

## Methods

### Overview of Study Design

**Fig. 1:**
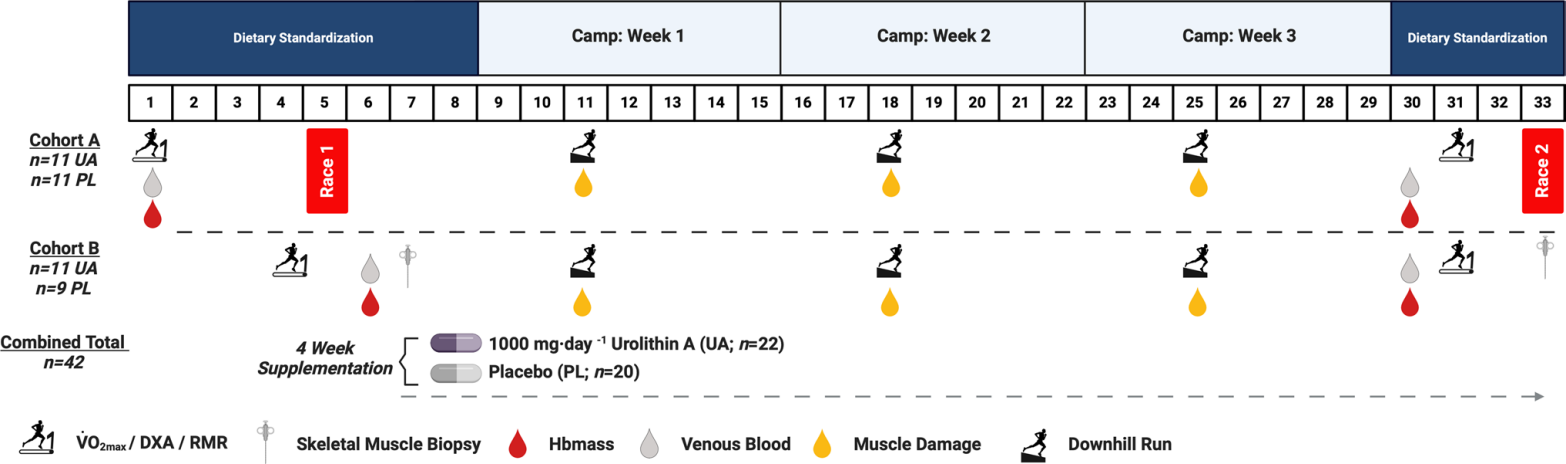
Study schematic. Forty-two highly trained male distance runners participated in this 5-week double-blind placebo-controlled clinical trial. Athletes were randomized to receive either 1000 mg·day^−1^ urolithin A (UA, *n* = 22) or placebo (PL, *n* = 20) for 4 weeks while completing an intensified 3-week training camp performed at low altitude (~ 1700–2200 m)

This double-blind, placebo-controlled parallel-group study was conducted within a residential training camp environment where athletes were supervised for all meals and training sessions. The study consisted of a 5-week camp including a 3-week structured altitude training block (Fig. 1). During week 1, athletes were housed at the Australian Institute of Sport (Canberra, ACT, Australia), where they undertook a battery of tests to identify baseline characteristics including submaximal running economy and $\dot{V}{\text{O}}_{{{\text{2max}}}}$, body composition (assessed via dual-energy X-ray absorptiometry, DXA), and hemoglobin mass (Hbmass) and had a resting venous blood sample taken. Participants in Cohort A completed a 3000 m time trial (TT) on a synthetic rubberized track (*n* = 22 total; *n* = 11 UA, *n* = 11 placebo; PL), while Cohort B (*n* = 20; *n* = 11 UA, *n* = 9 PL) had skeletal muscle biopsies taken to determine the impact of UA supplementation on global protein expression and in situ mitochondrial respiration in permeabilized muscle fiber bundles (PmFB) using high-resolution respirometry. All testing during this period was performed while following a standardized diet of high energy and carbohydrate (CHO) availability (220 kJ·day^−1^, 8.5 g·kg^−1^ body mass·day^−1^ CHO). Upon completion of baseline testing, athletes within each cohort (i.e., Cohort A and Cohort B) were pair-matched on the basis of $\dot{V}{\text{O}}_{{{\text{2max}}}}$, lifetime running personal bests, and training volume and randomized using a random number generator in a 1:1 ratio to receive either 1000 mg·day^−1^ UA or placebo (PL; composed of the same inert excipients as the active treatment) for 4 weeks. Pair-matching was done to limit differences between groups (UA versus PL) at baseline. Randomization was performed by a clinical trial manager external to the research team. Both the UA and PL supplements were provided in identical packaging containing visually identical soft gel capsules. Participants consumed 4 × 250 mg capsules (1000 mg total) each morning on an empty stomach with a glass of water. During the 3-week structured training camp, participants were housed at 1800 m elevation (Perisher Valley, NSW, Australia) with training sessions performed at low-to-moderate altitudes (1700–2200 m, Snowy Mountains, Australia).

### Participants

A total of 44 highly trained (tier 3–4 [21]) male middle- and long-distance runners were recruited between March 2021 and October 2022 to participate in this study. To be eligible, participants were required to be currently running > 70 km·week^−1^, have a $\dot{V}{\text{O}}_{{{\text{2max}}}}$ > 60 mL·kg^−1^·min^−1^ and have an official 3000 m personal best time of less than 9:00 (Cohort A; performance arm) or 10:00 min (Cohort B; muscle biopsy arm), and agree to participate in the training camp. One participant was lost to follow-up (did not report for baseline testing), and one participant withdrew with severe illness partway through the investigation, unrelated to the treatment. Their data were not included in final analysis, leaving a final sample size of 42 (CONSORT diagram, Supplementary Fig. 1). Subject characteristics are presented in Table 1. Ethics approval was obtained from the Australian Catholic University’s Human Research Ethics Committee (2021-36HC), and the study was prospectively registered as a clinical trial (NCT04783207). Comprehensive details of the study protocol were explained orally and provided in writing prior to athletes providing their written informed consent. All procedures conformed to the standards set by the Declaration of Helsinki.

**Table 1:** Subject characteristics

Characteristic	Placebo (*n* = 20)		Urolithin A (*n* = 22)	
	Pre	Post	Pre	Post
Age (year)	25.7 ± 1.3		28.7 ± 1.6	
Average Weekly Running Volume (km)	107.0 ± 6.1		94.3 ± 4.6	
3000 m Personal Best (mm:ss.ms)	8:57.4 ± 00:07.5		8:48.4 ± 00:07.8	
Body Mass (kg)	66.9 ± 1.4	67.5 ± 1.4	67.1 ± 1.6	67.7 ± 1.5
Fat Free Mass (kg)	59.2 ± 1.2	59.4 ± 1.2	59.8 ± 1	60.4 ± 1.3
Lean Mass (kg)	56.2 ± 1.1	56.3 ± 1.1	56.6 ± 1.4	57.0 ± 1.3
Fat Mass (kg)	8.0 ± 0.4	8.4 ± 0.4	7.8 ± 0.4	7.9 ± 0.4
Running Economy (@16 km·h^−1^, mL·kg^−1^·km^−1^)	190.8 ± 3.0	196.2 ± 3.1	192.7 ± 2.1	195.9 ± 2.2

Data are means ± SEM. No statistical differences were detected between groups at baseline (Pre) or following the intervention (Post)

### Assessments

#### Body Composition

At baseline and following the 3-week altitude camp, all athletes undertook a DXA assessment for an estimation of body composition (iDXA, GE Healthcare, Milwaukee, WI). These measurements were undertaken according to the Best Practice Protocols of the Australian Institute of Sport; participants reported in the early morning in an overnight fasted and rested state, while the same DXA technician positioned participants and analyzed all images (enCore v18, GE Healthcare). The test–retest technical error of measurement for the iDXA at our center is 0.65% for total mass, 0.95% for lean mass, 1.82% for fat mass, and 0.47% for bone mass.

#### Hemoglobin Mass

The erythropoietic response to altitude exposure and UA supplementation was assessed via measurement of total hemoglobin mass (Hbmass) using the optimized 2-min carbon monoxide (CO) rebreathing method. Briefly, a CO bolus (1.2 mL·kg^−1^ body mass) was rebreathed with 4 L 100% oxygen through a glass spirometer for 2 min. Carboxyhemoglobin (HbCO) concentration of capillary blood was measured in quintuplet before and 7 min post CO ingestion using an OSM 3 hemoximeter (Radiometer, Copenhagen, Denmark). Hbmass was calculated from the mean change in HbCO as described previously [22]. Hbmass was assessed at baseline and following the completion of the 3-week altitude training camp.

#### Incremental Exercise Testing

Prior to and following the altitude camp, all participants completed an incremental exercise test to exhaustion on a custom-built motorized treadmill (Australian Institute of Sport, Bruce, Australia) to determine submaximal running economy and $\dot{V}{\text{O}}_{{{\text{2max}}}}$. This test commenced 2 h after the intake of a standardized meal providing 2 g·kg^−1^ body mass CHO. A self-selected warm-up of 10 min duration preceded the test, which was maintained across trials. Running economy was assessed during four submaximal stages, each lasting 4 min and increasing in speed by 1 km·h^−1^ followed by 1 min standing rest. Starting speeds were selected at either 14 (Cohort B) or 16 km·h^−1^ (Cohort A) on the basis of each individual’s capacity, increasing to 17 or 19 km·h^−1^ at the final stage. Heart rate (HR) was measured continuously throughout the test (Polar Heart Rate Monitor, Polar Electro, Kempele, Finland). Expired gas was collected and analyzed using a custom-built indirect calorimetry system [14], with the final 60 s of gas collected accepted as steady state and used to calculate the respiratory exchange ratio (RER) and O_2_ uptake. Upon completion of the final submaximal stage, participants rested for 5 min before completing a ramp (speed and then gradient) test to volitional fatigue. The incremental test commenced at either 13 (Cohort B) or 15 km·h^−1^ (Cohort A) and was increased by 0.5 km·h^−1^ every 30 s until the speed corresponding to the individual’s final submaximal stage was reached (17 or 19 km·h^−1^), with treadmill gradient increased by 0.5% every 30 s thereafter until exhaustion. Expired gas was collected and analyzed throughout, and maximal HR recorded upon completion of the test. RER was calculated from steady-state expired gases collected over 1-min periods during the submaximal economy and $\dot{V}{\text{O}}_{{{\text{2max}}}}$ protocol. Rates of CHO and fat oxidation (g·min^−1^) were calculated as described previously [23]. Briefly, $\dot{V}{\text{O}}_{{2}}$ and $\dot{V}{\text{CO}}_{{2}}$ values were used to calculate substrate oxidation rates using nonprotein RER values [24]. These equations are based on the premise that $\dot{V}{\text{O}}_{{2}}$ and $\dot{V}{\text{CO}}_{{2}}$ accurately reflect tissue O_2_ consumption and CO_2_ production, and that indirect calorimetry is a valid method for quantifying rates of substrate oxidation in well-trained athletes during strenuous exercise of up to 85% of $\dot{V}{\text{O}}_{{{\text{2max}}}}$ [25].

#### 3000 m Race Performance

Prior to (Race 1) and following (Race 2) the altitude camp, participants in Cohort A completed a 3000 m time trial (TT) on a synthetic 400-m outdoor athletics track (Canberra, ACT, Australia). Race performance was individually hand-timed for each participant by a member of the research team, with elapsed time for each lap being announced during the race. Each race was preceded by a self-selected warm-up of ~15 min duration, which was maintained across trials for each participant. Capillary blood samples were collected immediately prior to and following the completion of the race for blood lactate analysis, with RPE (Borg Scale, 6–20) assessed at the same time points. Additional samples were collected for analysis of circulating markers of inflammation and muscle damage as outlined below.

#### Downhill Running Bout

To induce muscle damage, participants completed a bout of downhill running on Monday morning of each week at the altitude camp. The run consisted of a self-selected warm-up that was replicated across trials, followed by a ~4.5-km run on an asphalt surface with an average decline of 4%. Participants were instructed to run this as hard as possible and upon completion were driven back to the athlete residence for collection of capillary blood samples for assessment of inflammation and muscle damage markers as outlined below. Training in the day prior to and following the completion of the downhill run was replicated each week.

#### Circulating Markers of Inflammation and Muscle Damage

Capillary blood samples were collected from each participant to assess both CK and CRP in response to the 3000 m TT and the downhill runs. For the TT, samples were collected prior to the warm-up (0 h), as well as 1 and 24 h post race. For downhill runs, samples were collected in the morning prior to commencing exercise (0 h), as well as 1, 24, and 36 h post exercise. Blood collection required athletes to place a hand in a bucket of warm water for ~5 min to increase blood circulation, after which the hand was dried, and an incision made into a fingertip using a lancet. A 1 mL capillary blood sample was collected into a serum separator tube (Minicollect, Greiner Vacuette, Austria), which was left to clot for 30 min before being centrifuged at 1500* g* for 10 min. Serum was then divided into cryotubes and frozen at −80°C for batch analysis. CK and CRP were analyzed using a COBAS Integra 400 automated biochemistry analyzer (Roche Diagnostics, Rotkreuz, Switzerland).

#### Skeletal Muscle Biopsy Collection

Participants in Cohort B arrived at the laboratory following the consumption of a standardized meal having abstained from caffeine, alcohol, and exercise for the preceding 24 h. Local anesthetic (1% lignocaine hydrochloride in saline; McFarlane; Surrey Hills, Victoria Australia; 11037-AS) was administered to the vastus lateralis, after which two percutaneous skeletal muscle biopsies were collected using a Bergstrom needle modified with suction. A portion of the first biopsy was used for the preparation of permeabilized muscle fiber bundles (see below), while the second sample was immediately snap-frozen in liquid nitrogen and stored at −80°C for subsequent analysis.

#### Preparation of Permeabilized Fibers

A small portion of each biopsy was placed in ice-cold BIOPS (50 mM MES, 7.23 mM K_2_EGTA, 2.77 mM CaK_2_EGTA, 20 mM imidazole, 0.5 mM DTT, 20 mM taurine, 5.77 mM ATP, 15 mM PCr, and 6.56 mM MgCl_2_·H_2_O; pH 7.1) and separated under a microscope into bundles using fine-tipped forceps as described previously [26]. Fiber bundles were then treated with 30 μg·mL^−1^ saponin for 30 min at 4°C, then washed for 15 min in MiR05 respiration buffer (0.5 mM EGTA, 10 mM KH_2_·PO_4_, 110 mM sucrose, and 1 mg·mL^−1^ fatty acid-free bovine serum albumin (BSA); pH 7.1). Measurements of O_2_ consumption were performed using high-resolution respirometry (Oxygraph-2K, Oroboros Instruments, Innsbruck, Austria) at 37°C in the presence of 25 μM blebbistatin as previously described [27]. Pyruvate supported respiration was initiated with 10 mM pyruvate and 2 mM malate (PM), followed by 5 mM ADP (+ ADP), 10 mM glutamate (+ G), and 10 mM succinate (+ S). Lipid supported respiration was initiated with 0.2 mM octanoyl-carnitine and 0.5 mM malate (+O-Carnitine), followed by 2.5 mM ADP (+ ADP), and 10 mM succinate (+ S). Both protocols were concluded by adding 10 μM cytochrome *c* to assess mitochondrial membrane integrity. Experiments with a cytochrome *c* response greater than 10% were omitted from final analysis.

#### Muscle Proteomics Sample Preparation

Human muscle samples were homogenized and denatured using a urea-based proprietary denaturing buffer (Biognosys’ Denature Buffer). Samples were homogenized in Biognosys’ SDS Lysis Buffer using a Precellys Evolution homogenizer. Lysates were further prepared on a Hamilton Microlab STAR liquid handling system according to Biognosys’ standard operating procedures. Protein concentrations were measured with a BCA assay (Pierce, Thermo Fisher). Per sample, 70 µg of protein were reduced, alkylated, and digested to peptides using trypsin (Promega, 1:50 protease to total protein ratio) at 37°C. Peptides were desalted using an HLB µElution plate (Waters) and dried down. Peptides were resuspended in 1% acetonitrile/0.1% formic acid in water and spiked with Biognosys’ iRT kit calibration peptides. Peptide concentrations were determined with a microBCA assay (Pierce, Thermo Fisher).

For DIA LC–MS/MS measurements, 3.85 µg of peptides per sample were injected on an in-house packed reversed phase column on a ThermoScientific Vanquish Neo UHPLC nano-liquid chromatography system connected to a ThermoScientific Orbitrap Exploris 480 mass spectrometer equipped with a NanosprayFlex ion source and a FAIMS Pro ion mobility device (ThermoScientific). LC solvents were A: water with 0.1% FA; B: 80% acetonitrile, 0.1% FA in water. The nonlinear LC gradient was 1–50% solvent B for 172 min followed by a column washing step at 90% B for 5 min, and a final equilibration step of 1% B for one column volume at 64°C with a flow rate set to ramp from 500 to 250 nL·min^−1^ (min 0: 500 nL/min, min 172: 250 nL/min, washing at 500 nL/min). The FAIMS-DIA method consisted per applied compensation voltage of one full range MS1 scan and 34 DIA segments as described previously [28]. For whole-proteome analysis, DIA mass spectrometric data were analyzed using the software Spectronaut (version 17.1, Biognosys) with the default settings, including a 1% false discovery rate control at PSM, peptide, and protein level, allowing for two missed cleavages and variable modifications (N term acetylation, methionine and proline oxidation, ammonia loss, deamidation (NQ)). A human UniProt fasta database (*Homo sapiens*, 2023 01 01) was used, the default settings were used for the library generation. HRM mass spectrometric data were analyzed using Spectronaut software (Biognosys, version 17.1). The false discovery rate on peptide and protein level was set to 1% on experiment level, and data were filtered using row-based extraction. The direct DIA spectral library generated in this project was used for the analysis. The HRM measurements analyzed with Spectronaut were adjusted using global normalization to median intensity of proteins identified in all runs (sparse).

#### Differential Protein Expression Analysis

For testing of differential protein abundance comparing baseline to post-treatment for each group, protein intensities for each protein were analyzed using a two-sample Student’s *t* test (simple model). To assess protein expression changes between baseline and post-UA treatment visits while accounting for placebo effects, a linear mixed model framework was employed using the msqrob2 package [29]. Raw protein intensity values underwent log transformation followed by normalization using the median-center method. The model was formulated as follows: *treatment* + *visit* + *treatment:visit,* where treatment represents either UA or placebo, and visit indicates baseline or post-treatment visit. Visit was treated as an interaction term in the model. To assess the mean log_2_ expression between post-UA and baseline, corrected for Placebo effects, the contrast “(Intercept) + post + UA:post” was employed, with the respective statistical test being “*post* + *UA:post* = *0*.” Volcano plots were generated using − log_10_ of the nominal *p*-value (*y*-axis) and log_2_ fold change (*x*-axis) from the comparison of post-UA treatment versus baseline controlling for PL. Proteins with an absolute log_2_ fold change greater than 0.25 and a nominal *p* value less than 0.05 were considered significantly regulated. The top 10 proteins by [rank | *p* value | log_2_ fold change] were then labeled.

#### Gene Set Enrichment Analysis

For gene set enrichment analysis (GSEA), the cellular components (CC) collection of the Gene Ontology database (GO CCs) was utilized for both simple and linear mixed models. Enrichment analysis was performed separately for the “simple” and “robust regression” models using the R package ClusterProfiler [30]. Log_2_ fold change values between visits served as the protein ranking metric. Subsequently, the enrichment of GO CC gene sets among up- or downregulated proteins was statistically tested. The minimum and maximum gene set sizes were set to 10 and 500, respectively. Adjusted *p* values were determined using previously described methods [31]. Terms with an adjusted *p* value ≤ 0.05 were considered statistically significant and further characterized based on the respective normalized enrichment score (NES) sign as either activated (NES > 0) or repressed (NES < 0). Visualizations included Venn diagrams to depict common and unique activated/repressed GSEA GO CCs between placebo and UA comparisons, dot plots of protein ratio, and paired boxplots of normalized protein expression values, highlighting top enrichment proteins within specific terms, specifically the “mitochondrial protein-containing complex” GO CC term in the “robust regression” analysis.

#### Immunoblotting

Muscle tissues were lysed in denaturing buffer as described above with added protease and phosphatase inhibitor cocktails (Thermofisher, Waltham, MA, USA). Samples were sonicated for 20 min using an ultrasonic bath (Branson 1510) and centrifuged at 13000 RPM for 20 min at 4°C. Protein concentrations from collected supernatants were determined via DC protein assay (Bio-Rad Hercules, CA, United States, 500-0112). Lysates were eluted in 5 × Laemmli buffer (Bio-Rad, 1610747), and 10 μg of each sample was separated by SDS-PAGE (Bio-Rad, 4568086) and transferred onto PVDF membranes (Bio-Rad, 1704156). Membranes were washed in Tris-buffered saline containing 0.05% Tween 20 (TBS-T) and blocked for 1 h with 5% bovine serum albumin (Panobiotech, PANP06-1391100). The following primary antibodies were incubated overnight diluted at 4°C diluted in blocking buffer: Phospho-Parkin (Biorbyt, orb312554, 1:1000), total Parkin (Santacruz, sc-32282, 1:1000), OXPHOS Antibody Cocktail (Abcam, ab110413, 1:2000), and total 4-hydroxynonenal (Abcam, ab46545, 1:1000). After washing with TBS-T, membranes were incubated with secondary antibody (goat–anti-mouse, Abcam 10461444 and goat–anti-rabbit, Abcam 1051204-4, 1:10,000) for 1 h at room temperature. Membrane proteins were detected by enhanced chemiluminescence and the Chemidoc MP imaging system (Bio-Rad). The volume density of each target band was quantified using Bio-Rad Image Lab and normalized to total protein in each lane using stain-free imaging technology using Image Lab software (version 6.1, Bio-Rad), as previously described [32].

#### Citrate Synthase Activity

Muscle samples were lysed in homogenization buffer containing 50 mM Tris–HCl (pH 7.5), 1 mM EDTA, 1 mM EGTA, 10% glycerol, 1% Triton-X, 50 mM sodium fluoride, 5 mM sodium pyrophosphate with cOmplete Protease Inhibitor Cocktail, and PhosSTOP phosphatase inhibitor (Sigma-Aldrich, St. Louis, MO, USA). Samples were centrifuged at 16,000 *g* for 30 min at 4°C and protein concentration was determined in triplicate via bicinchoninic acid protein assay (Pierce, Rockford, IL, USA), against bovine serum albumin standards (Sigma–Aldrich). Samples were freeze-thawed 3 × in liquid nitrogen to disrupt cellular membranes prior to use in the assay, and then centrifuged at 900 *g* for 10 min at 4 °C. Citrate synthase (CS) activity was determined in triplicate on a microplate by adding the following: 5 μL of muscle homogenate at a concentration of 2 µg·µL^−1^, 40 μL of 3 mM acetyl coenzyme A, and 25 μL of 1 mM 5,5′-dithiobis(2-nitrobenzoic acid) to 165 μL of 100 mM Tris buffer (pH 8.3) kept at 30°C. After the addition of 15 μL of 10 mM oxaloacetic acid, the plate was immediately placed in a plate reader (SpectraMax Paradigm, Molecular Devices, Sunnyvale, CA, USA) at 30°C. Absorbance was read at 412 nm and was recorded every 15 s for 3 min after 30 s of linear agitation. CS activity was normalized to protein content determined via BCA assay and is reported as µmol·min^−1^·g^−1^ protein.

#### Evaluation of Urolithin A Pharmacokinetics

Pharmacokinetics of the main urolithin A metabolite UA-glucuronide were analyzed in a subset of participants (*n* = 6 UA, *n* = 4 PL) using dried blot spots (DBS) collected on blood collection cards (Whatman Filter Paper 903 spots card). Fingertip capillary blood samples were collected prior to consuming the first dose of either UA or PL (0 h), and then at 1, 2, 6, and 24 h post ingestion. The 24 h timepoint was collected prior to the ingestion of the subsequent dose of either UA or PL. Each sample consisted of three to four blood spots containing ~ 20–40 µL of whole blood that were spotted on DBS cards. The cards were subsequently dried and stored in sealable biohazard foil bags containing desiccant at room temperature until analysis. Analysis and quantification of UA glucuronide was performed using liquid chromatography coupled to mass spectrometry as described previously [33]. The quantification of UA glucuronide was performed by column separation with reversed phase liquid chromatography followed by detection with triple-stage quadrupole MS/MS in the selected reaction monitoring mode. The concentration of UA glucuronide was calculated using the internal standardization method, with a quantification limit of 5.00–5000 ng mL^−1^. The acquisition and processing of data was performed using LCquan version 2.5.6 and Xcalibur version 2.0.7 (Thermo Fisher Scientific).

### Statistical Analysis

To account for two primary endpoints (CK for muscle damage and 3000 m TT performance), a hierarchical order for testing null hypotheses was selected a priori in the study protocol, including a clear specification of the set of hypotheses that need to be significant [34]. For this study, a change in CK was tested first and then race performance was subsequently tested as a co-primary endpoint. The 95% CIs for treatment differences and corresponding nonadjusted *p*-values were calculated. A 5% significance level (*α* = 0.05 or two-sided *p* < 0 0.05) was applied for the comparison of treatment groups. *p*-values represent the interaction between treatment and time. If significance was detected, a Bonferroni post hoc was applied. Model assumptions for the repeated measures mixed-effects model were also assessed. Residuals were visually inspected for normality and homoscedasticity using diagnostic plots. Point estimates, 80% CIs, and 95% CIs were extracted from the analysis of covariance model. All analyses were performed using SAS^®^ version 9.4 with SAS Enterprise Guide version 8.3. For within-group analysis, a comparison of means from baseline was performed using a two-tailed Welch’s *t* test. Total area under the curve (tAUC) was calculated using the Time Response Analyser [35] to assess CK and CRP responses to exercise over time. Statistical analysis was conducted on raw data, with supporting descriptive statistics (% change) and effect sizes (Cohens *d*) included in text where appropriate. Effect sizes using Cohen’s *d* were calculated using the difference between group means divided by the pooled standard deviation, and interpreted using thresholds of > 0.2, > 0.5, and > 0.8 for small, moderate, and large effect sizes, respectively [36]. The pooled standard deviation was computed as the square root of the weighted average of the two groups’ variances, accounting for sample size. A formal power calculation was not done prior to commencing the study given the lack of relevant literature assessing supplements targeting mitochondrial function and subsequent impact on running performance related outcomes in well-trained individuals. Sample size was therefore estimated based on similar studies exploring mitochondrial targeting supplements and endurance performance [37, 38]. Figures were produced using GraphPad Prism (version 10.4.1, GraphPad Software Inc., La Jolla, CA, USA). The schematics in Figs. 1 and 4A were created using BioRender.com.

## Results

### Muscle Damage and Inflammation Responses to Exercise

**Fig. 2:**
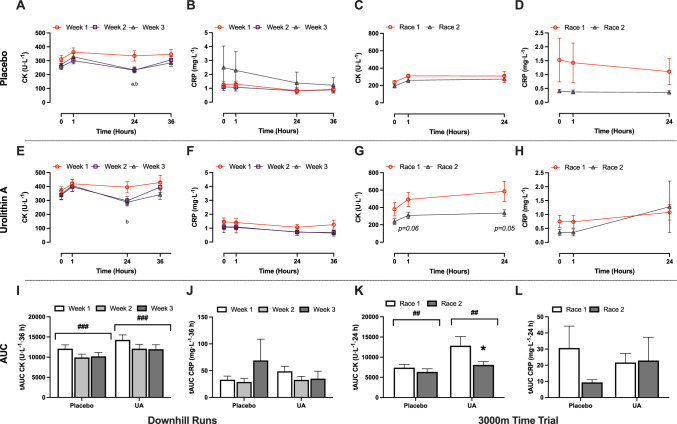
Capillary blood samples were collected from all participants (UA, *n* = 22; PL*, n* = 20) following a weekly downhill run and assessed for markers of muscle damage (creatine kinase, CK; **A**, **E**, **I**) and inflammation (C-reactive protein, CRP; **B**, **F**, **J**). These markers were also assessed prior to and following the 3000 m time trial (*n* = 11 per group, CK; **C**, **G**, **K**, CRP; **D**, **H**, **L**). Data are presented as mean ± SEM. **A**–**H** statistical difference ^a^*p* < 0.05 Week 2 versus Week 1, ^b^*p* < 0.05 Week 3 versus Week 1. **I**–**L** main effect of time, ^*##*^*p* < 0.01, ^*###*^*p* < 0.0001. Statistically different within group compared with baseline, **p < 0.01, ***p < 0.0001

CRP and CK were measured following the downhill running bout and the 3000 m TT and are summarized in Fig. 2. Within the PL group, serum CK levels measured 24 h after the downhill run were lower in Week 2 (*p* = 0.029, *d* = 0.72) and Week 3 (*p* = 0.042, *d* = 0.66) compared with Week 1 (Fig. 2A). Within the UA group, a significant decrease in CK was observed 24 h post exercise in Week 3 relative to Week 1 (*p* = 0.037, *d* = 0.65; Fig. 2E). CK levels returned to baseline 36 h post-exercise for both PL and UA. Analysis of 36 h total area under the curve (tAUC) for CK post downhill run (Fig. 2I) showed a significant effect of time (*p* < 0.0001), but no effect of treatment (*p* = 0.137) or interaction (time × treatment, *p* = 0.891).

There were no significant changes in absolute CK concentrations in the PL group at any time point during Race 2 compared with Race 1 (Fig. 2C). However, there was a significant time × treatment interaction effect at both 1 h (*p* = 0.008) and 24 h (*p* = 0.035). While the decreases in the UA group between Race 1 and Race 2 did not reach statistical significance, a large and potentially meaningful effect was found at both 1 h (*p* = 0.056, *d* = 0.87) and 24 h (*p* = 0.055, *d* = 0.87) (Fig. 2G). Analysis of tAUC (Fig. 2K) revealed a significant main effect of time (*p* = 0.005) and a time × treatment interaction (*p* = 0.0489), as CK levels were lower following Race 2 in the UA group (*p* = 0.0016, *d* = 0.81), but not in PL (*p* = 0.450, *d* = 0.40). No significant changes in serum CRP concentrations were detected in either treatment group in response to either the 4.5-km downhill runs (Fig. 2B, F) or TT (Fig. 2D, H), or in calculated tAUC (Fig. 2J, L).

### 3000 m TT Running Performance and Whole-Body Physiological Adaptations

**Fig. 3:**
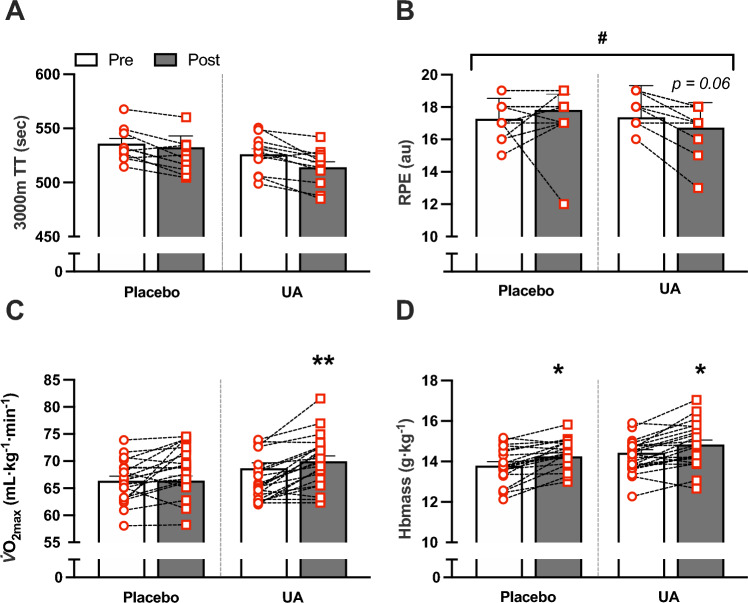
Whole-body physiological outcomes were measured at baseline (Pre) and following the camp and supplementation period (Post), including 3000 m time trial (TT) performance (**A**) and post-race rate of perceived exertion (RPE; **B**), as well as aerobic capacity ($\dot{V}{\text{O}}_{{{\text{2max}}}}$; **C**) and hemoglobin mass (Hbmass; **D**). Data are presented as mean ± SEM. Differences between groups are indicated by brackets, ^#^*p* < 0.05. Statistically different within group compared to baseline, *p < 0.05, **p < 0.01

While TT performance improved in both groups compared with baseline (UA; 513.9 ± 5.2 versus 526.0 ± 5.2 s, PL; 532.5 ± 10.5 versus 535.9 ± 4.9 s), this did not reach statistical significance (UA; *p* = 0.116, *d* = 0.70, PL; *p* = 0.771, *d* = 0.13, Fig. 3A). RPE was numerically lower following the completion of Race 2 compared with Race 1 in athletes supplementing with UA (Fig. 3B, p = 0.064, *d* = 0.84), with a significant time × treatment effect detected between groups (*p* = 0.020). Although no interaction effect was detected for aerobic capacity (*p* = 0.138), the UA group showed a large within-group increase in $\dot{V}{\text{O}}_{{{\text{2max}}}}$ (66.4 ± 0.8 to 70.0 ± 1.0 mL·kg^−1^·min^−1^, *p* = 0.009, *d* = − 0.83), compared with a moderate change in the PL group (66.4 ± 0.9 to 68.7 ± 1.0 mL·kg^−1^·min^−1^, *p* = 0.098, *d* = − 0.55). Hbmass was similarly increased in both UA and PL groups following the training camp (14.2 ± 0.2 to 14.8 ± 0.2 g·kg^−1^, *d* = − 0.63 and 13.8 ± 0.2 to 14.4 ± 0.2 g·kg^−1^, *d* = − 0.80 respectively, both *p* < 0.05 versus baseline, Fig. 3D), with no difference between groups (*p* = 0.877). There were no changes in body composition (total body mass, lean mass, fat, or fat free mass) or submaximal running economy (assessed at 16 km·h^−1^) following the camp, and no differences were detected between groups (Table 1, all *p* > 0.05).

### Skeletal Muscle Proteome

**Fig. 4:**
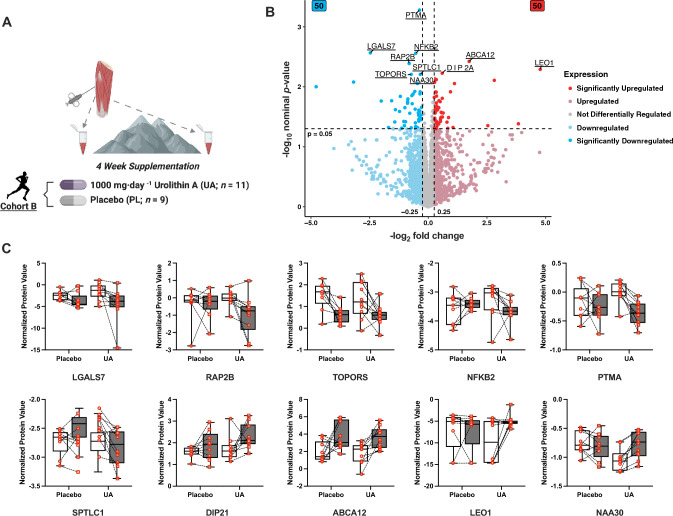
Skeletal muscle biopsy samples were collected at rest from a subset of participants (Cohort B) following 4 weeks supplementation with either UA (*n* = 11) or PL (*n* = 9) including a 3-week training camp performed at 1,700–2,200 m elevation (**A**). Muscle samples were subjected to liquid chromatography with tandem mass spectrometry (LC–MS/MS) to identify and quantify changes in the skeletal muscle proteome in response to the intervention. Over 6600 + proteins were detected with the volcano plot (**B**) showing the median log_2_ fold change (*x*-axis) plotted against the − log_10_ nominal *p* value (*y*-axis). Proteins with an absolute log_2_ fold change greater than 0.25 and a nominal *p* value less than 0.05 were considered significantly regulated, including 50 significantly upregulated, and 50 significantly downregulated. The top 10 proteins by [rank | *p* value | log_2_ fold change] are labeled. Boxplot quantification of these targets (**C**) representing the interquartile range with median, with whiskers for minimum and maximum non-outlier values. Individual points for each subject are log_2_ transformed and median-centered protein intensity values

Proteomic analysis was performed in individuals from Cohort B (UA; *n* = 11, PL; *n* = 9) from skeletal muscle biopsy samples collected prior to and following the 4-week intervention (Fig. 4A). We detected 6600 + proteins, with 50 proteins significantly upregulated and 50 proteins significantly downregulated following UA supplementation when normalized relative to PL (absolute log_2_ fold change greater than 0.25 and a nominal *p* value less than 0.05, Fig. 4B). Two proteins (ZC3H11B and SCGB1D2) were ruled as outliers as a result of their unusually high log_2_ fold changes (9.2 and 7.1, respectively), and relatively low expression across the cohort (Supplementary Fig. 3). The top 10 proteins by rank included Disco-interacting protein 2 homolog A (gene name DIP2A), ATP binding cassette subfamily A member 12 (ABCA12), RNA polymerase-associated protein (LEO1), and *N*-acetyltransferase 12 (NAA30). Downregulated proteins included nuclear factor kappa B subunit 2 (NFκB2), Ras-related protein Rap-2b (RAP2B), E3 ubiquitin-protein ligase Topors (TOPRS), galectin-7 (LGALS7), serine palmitoyltransferase 1 (SPTC1), and prothymosin alpha (PTMA) (Fig. 4C).

**Fig. 5:**
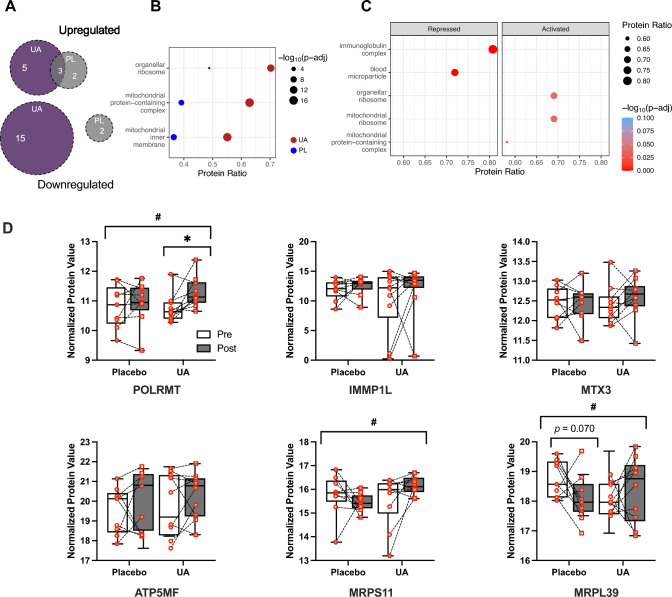
Gene ontology (GO) pathway analysis revealing 5 distinct upregulated and 15 downregulated with daily UA supplementation (**A**), with only 3 shared upregulated pathways compared with PL. These common pathways consisted of proteins associated with organellar ribosomes, mitochondrial protein-containing complexes, and mitochondrial inner membrane (**B**). Relative to PL, there was a significant upregulation of these pathways with UA, and a downregulation of proteins associated with the immunoglobulin complex and blood microparticles (**C**). Targets in these pathways were then validated (**D**); boxplot representing the interquartile range with median, with whiskers for 5–95^th^ percentiles. Individual points for each subject are log_2_ transformed and median centered protein intensity values. Open bars, baseline testing (Pre); filled bars, post-camp testing (Post). Differences between groups are indicated by brackets, ^#^*p* < 0.05. Statistically different within group compared with baseline, *p < 0.05, **p < 0.01

Gene Ontology (GO) pathway analysis revealed there were 5 distinct upregulated pathways and 15 distinct downregulated pathways induced by training combined with UA supplementation compared with the training camp alone (PL). Three upregulated pathways were shared by both treatment groups (Fig. 5A), consisting of proteins associated with organellar ribosomes, mitochondrial protein-containing complexes, and the mitochondrial inner membrane (Fig. 5B). Data indicate a stronger impact on mitochondrial pathways in the group training with UA supplementation compared with athletes in the PL group. Gene set enrichment analysis (GSEA) using the GO dataset revealed that proteins associated with mitochondrial protein-containing complexes, as well as organellar and mitochondrial ribosomes were the top hits significantly enriched in athletes consuming UA compared with PL (Fig. 5C, all *p* < 0.05), while proteins associated with immunoglobin complex and blood microparticles were significantly downregulated (*p* < 0.05). Validation of targets belonging to the upregulated pathways (Fig. 5D) showed statistically significant increases in the expression of proteins responsible for mitochondrial gene expression (POLRMT; *p* = 0.023 UA post versus baseline, *p* = 0.032 versus PL), and mitochondrial ribosome protein subunits (MRPS11; *p* = 0.012 versus PL, MRPL39; *p* = 0.001 versus PL).

### Substrate Oxidation, Skeletal Muscle Mitochondrial Content, and Function

**Fig. 6:**
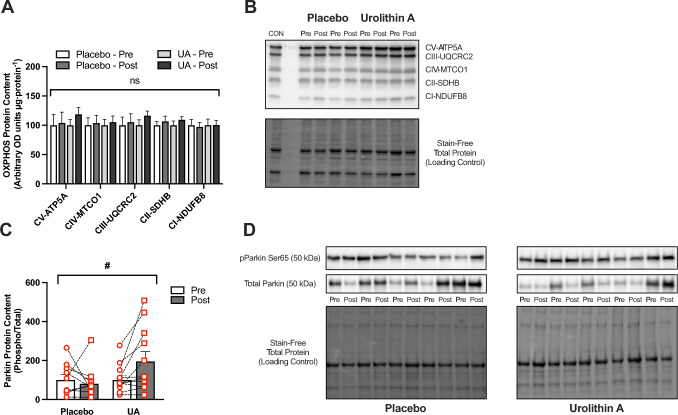
Quantified protein expression assessed in skeletal muscle biopsy samples collected from well-trained endurance athletes following a 4-week supplementation with either urolithin A (*n* = 11) or placebo (*n* = 9). Electron transport chain complexes (OXPHOS, **A**) and representative image and stain-free total protein (**B**). Quantified cell signaling responses (**C**) for mitophagy-associated pathways (total and phosphor-Parkin Ser65) with representative image and stain-free total protein (**D**). Data are mean ± SEM. Differences between groups are indicated by brackets, ^#^*p* < 0.05

Rates of whole-body substrate oxidation were calculated during submaximal exercise across the entire cohort (*n* = 42). Fat oxidation increased relative to baseline testing (Pre) during stage 3 (*p* = 0.021) and 4 (*p* = 0.039) in the PL group (Table 2), but there were no differences detected between treatment groups (UA versus PL, *p* > 0.05). Rates of CHO oxidation increased with increasing exercise intensity (Table 2), but there were no changes following the training camp or between groups (all *p* > 0.05). In support of these whole-body exercise-induced responses, we did not detect a difference in maximal lipid- or pyruvate-supported mitochondrial respiration in PmFB following the 3-week altitude training camp and supplementation with either UA or PL (all *p* > 0.05, Table 2). While citrate synthase activity increased in both groups, this did not reach significance (Table 2, 11 ± 2% increase in UA, *p* = 0.260; 16 ± 3% increase in PL, *p* = 0.262). There were also no changes in protein expression of the electron transport chain complexes (OXPHOS; Fig. 6A, B) following the camp, or between treatment groups. Although the increase in phosphorylation of the Ser65 site of Parkin relative to total Parkin protein content with UA supplementation was not statistically significant, the effect size was in the medium range, suggesting a potentially meaningful difference (Fig. 6C, D, *p* = 0.098, *d* = − 0.74) with a significant time × treatment effect detected between groups (*p* = 0.019).

**Table 2:** Assessment of substrate oxidation patterns and mitochondrial function

Characteristic	Placebo			Urolithin A		
	Pre	Post	*p* value	Pre	Post	*p* value
*Calculated fat oxidation rate during submaximal running (g·min*^*−1*^*)*						
Stage 1	0.48 ± 0.04	0.59 ± 0.05	0.103	0.47 ± 0.04	0.56 ± 0.05	0.203
Stage 2	0.27 ± 0.03	0.37 ± 0.05	0.082	0.27 ± 0.04	0.39 ± 0.05	0.080
Stage 3	0.12 ± 0.03	0.25 ± 0.05	0.021	0.16 ± 0.04	0.26 ± 0.05	0.143
Stage 4	0.04 ± 0.02	0.13 ± 0.04	0.039	0.08 ± 0.03	0.17 ± 0.05	0.113
*Calculated CHO oxidation rate during submaximal running (g·min*^*−1*^*)*						
Stage 1	2.95 ± 0.12	2.79 ± 0.13	0.376	3.15 ± 0.16	2.98 ± 0.13	0.422
Stage 2	3.82 ± 0.16	3.66 ± 0.13	0.434	3.94 ± 0.19	3.74 ± 0.16	0.427
Stage 3	4.65 ± 0.18	4.36 ± 0.18	0.262	4.68 ± 0.24	4.47 ± 0.19	0.494
Stage 4	5.31 ± 0.17	5.25 ± 0.20	0.822	5.26 0.25	5.27 ± 0.20	0.975
*Lipid supported mitochondrial respiration (pmol·s*^*−1*^*·mg ww*^*−1*^*)*						
+ O-Carnitine	7.41 ± 0.74	7.52 ± 0.74	0.918	8.99 ± 1.12	11.79 ± 3.12	0.499
+ ADP	29.36 ± 3.68	34.74 ± 3.01	0.272	37.55 ± 4.41	36.24 ± 3.18	0.808
+ ADP + Succinate	50.12 ± 5.01	60.12 ± 3.79	0.127	69.81 ± 10.11	57.84 ± 5.51	0.274
+ Cytochrome *c*	50.28 ± 5.68	61.24 ± 3.73	0.120	71.01 ± 10.15	59.56 ± 5.23	0.285
*Pyruvate supported mitochondrial respiration (pmol·s*^*−1*^*·mg ww*^*−1*^*)*						
+ Pyruvate + Malate	7.52 ± 0.55	8.31 ± 0.80	0.399	7.29 ± 0.61	8.69 ± 0.69	0.178
+ ADP	57.91 ± 8.15	59.32 ± 8.22	0.905	53.90 ± 10.80	57.93 ± 4.49	0.698
+ ADP + Glutamate	60.47 ± 8.42	61.71 ± 8.84	0.921	54.67 ± 10.95	61.18 ± 4.53	0.537
+ ADP + Glutamate + Succinate	83.36 ± 10.02	92.83 ± 12.26	0.565	69.45 ± 12.36	84.10 ± 6.83	0.276
+ Cytochrome* c*	83.35 ± 10.34	91.43 ± 12.14	0.624	68.63 ± 13.47	85.26 ± 7.07	0.247
*Citrate synthase activity (μmol·min*^*−1*^*·g*^*−1*^* protein)*						
	268.74 ± 59.07	303.81 ± 68.63	0.262	313.70 ± 52.75	342.96 ± 64.93	0.260

Submaximal running stages 1–4 were performed at 16–19 (Cohort A) and 14–16 (Cohort B) km·h^−1^. Indirect calorimetry data: UA, *n* = 22; PL, *n* = 20. Mitochondrial respiration data are normalized to tissue wet weight (ww): UA, *n* = 7–11; PL, *n* = 8–9. Citrate synthase activity: UA, *n* = 11; PL, *n* = 9. Data are means ± SEM

## Discussion

We evaluated the impact of 4 weeks of daily supplementation with UA on markers of recovery, aerobic capacity, mitochondrial function, and running performance in highly trained male distance runners following a training camp performed at 1700–2200 m altitude. Assessments of both lipid- and pyruvate-supported mitochondrial respiration were unaltered by either treatment or by the training intervention. However, proteomic analysis of skeletal muscle biopsy samples revealed that, relative to PL, daily UA supplementation upregulated proteins in pathways associated with organellar ribosomes, mitochondrial protein-containing complexes. In contrast, proteins associated with the immunoglobulin complex and blood microparticles were downregulated after UA treatment. While no interaction effect was detected for aerobic capacity, the percentage increase and calculated effect size for the change in $\dot{V}{\text{O}}_{{{\text{2max}}}}$ was larger in the UA group (5.4 ± 0.9%, *d* = − 0.83) compared with the PL group (3.6 ± 1.3%, *d* = − 0.55) despite both groups displaying similar increases in Hbmass (~4–5%). Collectively, these physiological changes highlight the success of the training camp in inducing adaptation to a robust training stimulus. Yet, despite these robust physiological adaptations, and in contrast to our hypothesis, we were unable to detect clear differences between treatments on 3000 m TT performance. While CK levels (an indirect measure for muscle damage) were not impacted by UA supplementation in response to a ~4.5 km downhill run, the 24-h tAUC was significantly reduced following a 3000 m TT in individuals supplementing with UA compared with PL (*p* < 0.0001), providing evidence of a beneficial effect of UA on recovery from exercise-induced muscle damage.

### UA Reduces Circulating Indirect Markers of Muscle Damage But Not Inflammation

A single bout of exercise is associated with an acute wave of inflammatory pathways and cytokine signaling [39], while exercise-induced structural damage to the myofibrils also induces an inflammatory response to instigate tissue repair and cellular remodeling [40]. This can result in an acute decrease in muscle function, including speed, power, strength, and economy of movement [41, 42], with full recovery taking several days depending on the severity of damage and the habitual training status of the individual [43]. Given previous work has demonstrated that UA decreases markers of inflammation [4], we characterized inflammatory responses and indirect markers of muscle damage via measurement of circulating CRP and CK levels following both a weekly downhill running session and a 3000 m TT performed on a synthetic running track. While we did not detect changes in CRP levels in response to either form of exercise, CK levels were elevated in all athletes ~1 h post exercise, returning to baseline 24–36 h later. This is consistent with previous reports demonstrating an increase in CK activity, oxidative stress, and greater apoptosis 24 and 48 h after a bout of downhill running in moderately trained individuals [44]. The reduction in CK tAUC in both treatment groups following the downhill runs in Week 2 and 3 relative to Week 1 suggests adaptation to this training stimulus as the camp progressed. In contrast, the 24 h tAUC for CK was significantly reduced following Race 2 compared with Race 1 in athletes supplementing with UA. It is unclear why greater differences in circulating CK levels were observed between groups following the TT compared with the downhill runs, where we would have anticipated greater eccentric loading. This may be due in part to the increased intensity of the race, as the average race pace intensity of a 3000 m in highly trained athletes is estimated to be ≥ 100% $\dot{V}{\text{O}}_{{{\text{2max}}}}$ [45], or due to the differences in footwear between modalities (e.g., racing in track spikes compared with performing the downhill run in cushioned running shoes). Indeed, differences in CK levels have previously been shown as a result of type, intensity, and duration of exercise [46]. The absence of significant increases in CRP in the current study may also reflect the relatively short duration of exercise (~8–20 min) and time course of measurements (24–36 h), which may have been insufficient to induce a response of similar magnitude as observed in previous studies [47]. Future work should consider expanding upon these findings by utilizing more comprehensive measurements of muscle damage and inflammation, including additional biochemical markers (e.g., lactate dehydrogenase, myoglobin, troponin, cytokine panels, etc.) as well as direct measurements of changes in muscle function (e.g., strength, power, range of motion, contractile function [48]), and following most strenuous and/or prolonged exercise.

High-altitude exposure (4100 m) induces oxidative stress in skeletal muscle [49], which may be associated with an increase in inflammation and impaired immune function [17]. Indeed, alterations in immunological parameters in response to both training and altitude exposure have been found in a group of Olympic-level swimmers during a training camp conducted at a similar altitude (~2100 m) [50]. We performed additional exploratory analysis on a panel of inflammation markers (interferon-γ, interleukin (IL)-1β, IL-2, Il-4. IL-6, Il-10, IL-12p70, IL-17A, and tumor necrosis factor-α) using resting serum samples collected from all athletes pre/post intervention however no differences were detected in between treatment groups or collection time points (Supplementary Fig. 4). This may be due to the fact that the samples were collected at rest following an overnight fast at lower altitudes (i.e., near sea level, ~ 500 m elevation). Additionally, some of the immune and inflammatory responses to altitude and intensified training may, in part, be attributed to decreased energy and/or carbohydrate availability [17]. Our training camp model employed an integrated support team of dietitians as well as a camp chef who provided all meals to participants, ensuring they were adequately fueled to support training demands. As a result, participants’ body mass and composition remained stable, which likely also contributed to significant hematological adaptations including the ~4–5% increase in Hbmass across the participant cohort. Nevertheless, the maintenance of energy availability coupled with the training status of our cohort may have limited our ability to detect major differences in circulating inflammatory responses and underpin the differences in our results compared with previous work [17, 39, 50].

In contrast to our analysis of blood biomarkers, skeletal muscle biopsy samples showed a significant reduction in proteins associated with inflammatory/pro-apoptotic pathways following UA supplementation including NFκB2, a subunit of the NFκB transcription factor complex; TOPRS, a E3 ubiquitin-protein ligase; LGALS7 a pro-apoptotic protein; and PTMA, a highly conserved acidic protein implicated in oxidative stress responses [51]. Pathway analysis also showed a reduction in immunoglobulin complex associated proteins detected in skeletal muscle in individuals relative to PL. When combined with the reduced CK tAUC observed following the 3000 m TT, these findings suggest that UA may facilitate post-exercise recovery in highly trained individuals by decreasing skeletal muscle inflammation.

### Impact on Mitochondrial Function

Many of the effects of UA in clinical human populations have been attributed to increased mitochondrial gene [5] and protein [4] expression, suggesting improved mitochondrial quality control via an upregulation of mitophagy [52]. However, the role of mitophagy in response to acute and chronic exercise in human skeletal muscle is not well understood [6]. While our skeletal muscle sampling timepoints limited us to resting measurements, we detected a significant mean increase between groups in the phosphorylation of Parkin, a E3 ubiquitin ligase in the PINK1/Parkin mitophagy pathway. Phosphorylation increases the activity of Parkin, leading to ubiquitination of protein targets on the outer mitochondrial membrane, autophagosome formation, and subsequent lysosomal degradation [53, 54]. However, we were unable to detect any downstream changes on mitochondrial quality control—as there was no change in traditional markers of mitochondrial content (citrate synthase activity and OXPHOS protein expression), or in maximal mitochondrial respiration. This is consistent with previous work showing no effect of 9–11-days exposure to high-altitude (≥ 3500 m) on mitochondrial function [55], whereas prolonged (28 days) exposure has had equivocal effects on mitochondrial capacity [56, 57] but may increase mitochondrial efficiency [56]. In contrast, assessment of changes to the proteome coupled with gene set enrichment analysis (GSEA) revealed that proteins associated with mitochondrial protein-containing complexes as well as organellar and mitochondrial ribosomes were significantly enriched in athletes consuming UA compared with PL. Furthermore, validation of targets belonging to these upregulated pathways demonstrate for the first time that UA induced an additional effect on pathways linked to mitochondrial protein expression beyond those induced by training alone.

### Aerobic Capacity is Increased but Endurance Performance is Unaltered

While no significant time × treatment interaction effect was detected for $\dot{V}{\text{O}}_{{{\text{2max}}}}$, a larger effect (*d* = − 0.83) was shown in the UA group compared with PL (*d* = − 0.55). This occurred despite Hbmass increasing in both treatment groups to a similar extent (~4–5%), suggesting additional non-hematological adaptations may underpin the augmented aerobic power in UA supplemented athletes as outlined above in our GSEA data. Furthermore, while the Hbmass–$\dot{V}{\text{O}}_{{{\text{2max}}}}$ relationship may uncouple following altitude training, there is a weak but significant correlation in which a 1% increase in Hbmass enhances aerobic capacity by ~0.6–0.7% [58], consistent with the $\dot{V}{\text{O}}_{{2}}$ changes (3.6 ± 1.3%) observed in the PL group. However, the increase in maximal aerobic capacity in the UA group (5.4 ± 0.9%) failed to result in a clear performance improvement.

The outcomes of races in elite sport are determined by very small margins, with < 1% often separating medalists in championship distance races. The coefficient of variation (CV) of performance in elite athletes in middle distance/distance events, which determines whether the observed effect of an intervention might cause a meaningful change in race outcomes, has been reported at ~1.1–1.6% [59, 60]. The performance CV of the current cohort (*n* = 22) was 1.5 ± 4.4% (7.6 ± 1.4 s, mean ± SEM). Therefore the ~2.3 ± 0.6% (12.1 ± 3.1 s, *d* = 0.70) improvement in the UA group likely represents a meaningful real-world improvement for highly trained athletes based on a smallest worthwhile change of ~3.6 s compared with the 0.6 ± 1.4% (3.4 ± 7.3 s, *d* = 0.13) change detected in the PL group [61]. However, we note that sports performance is underpinned by a complex array of factors other than physiological characteristics, including external/environmental conditions, psychological readiness, and tactical and pacing strategies. Previous research has demonstrated a significant negative impact of muscle damage on subsequent endurance running performance, which was also associated with an increased sense of effort [62]. Consistent with this, in the current study, we found a reduction in post-race rate of perceived effort alongside the decrease in post-race CK tAUC following UA supplementation. The combination of augmented aerobic capacity coupled with improved recovery and perception of effort may underpin the differences in race performance noted above.

### Potential Limitations

We note that there was considerable variability in plasma responses assessed in this study, which likely reflect the unique aspects of the research embedded training camp study design employed. Specifically, these camps reproduce the real-world responses of athletes who are engaging in regular daily structured training. In the current study, CK levels in the UA group were higher than the PL group in Race 1 and during the downhill runs. However, this was consistent across participants in all three camps, and researchers were blinded to treatment groups during both sample collection and analysis. Furthermore, individuals were pair-matched across treatment groups based on training volume, personal bests, and $\dot{V}{\text{O}}_{{{\text{2max}}}}$ to limit variability between groups. Given the reduction in CK levels in the UA is consistent with the reduction in proteins associated with inflammatory/pro-apoptotic pathways in skeletal muscle samples, we are confident our findings reflect a meaningful biological response. In the current study, different cohorts of participants were used for performance and collect skeletal muscle biopsies measures. This was done to maximize recruitment and retention of highly trained athletes, and to increase the feasibility of measuring blood time-course responses for CRP and CK post 3000 m TT without the confounding variable of skeletal muscle biopsies and remove the influence of prior exercise on mitochondrial respiration within the limited testing window pre/post camp. However, we note that this may increase variability in some results and therefore limit our power to detect smaller changes in outcome measurements.

### Conclusions and Future Directions

Collectively, our findings demonstrate that, while running performance was not further enhanced by UA supplementation, a potentially meaningful larger effect on $\dot{V}{\text{O}}_{{{\text{2max}}}}$ was observed alongside a downregulation of the inflammation response in muscle in highly trained endurance athletes undergoing short-term intensified training. Proteomic analysis revealed UA upregulated the expression of proteins associated with mitochondrial protein-containing complexes, and downregulated proteins associated with immunoglobulin complexes. However, skeletal muscle mitochondrial function assessed in permeabilized fibers was unaltered by training or UA supplementation. Future studies should expand upon these findings using more sensitive techniques and time points to elucidate possible connections between reduction in skeletal muscle damage observed in individuals supplementing with UA and changes in mitophagy. Our results suggest that UA may be an effective supplemental strategy to enhance recovery from exercise and could enhance performance through alternative mechanisms beyond alterations in mitochondrial function. As our supplementation period was only 4 weeks, future work should explore the impact of longer-term supplementation with UA in individuals and situations where physiological recovery processes may be challenged owing to high training demands and/or scenarios in which exercise induces more substantial muscle damage, while also exploring the impact of UA on cognitive function, perception, and pacing effort.

## Supplementary Information

Below is the link to the electronic supplementary material.

Supplementary Figure 1. CONSORT Diagram (PDF 35 kb)

Supplementary Figure 2. Pharmacokinetics of the main urolithin A metabolite UA-glucuronide (panel A) was analyzed in a subset of participants (n=6 UA, n=4 PL) using dried blot spots (DBS) collected on blood collection cards. Fingertip capillary blood samples were collected prior to consuming the first dose of either UA or PL (0 h), and 24 h post-ingestion, with 24 h total area under the curve (tAUC) calculated (panel B). Data are means±SEM, **p<0.01. (PDF 42 kb)

Supplementary Figure 3. Volcano plot (**A**) showing the median log_2_ fold change (x-axis) plotted against the –log_10_ nominal p-value (y-axis) including outliers (SCGB1D2, ZH3H11B). Proteins with an absolute log_2_ fold change greater than 0.25 and a nominal *p*-value less than 0.05 were considered significantly regulated. The log_2_ fold change for ZC3H11B and SCGB1D2 was 9.2 and 7.1, respectively despite sparse expression across samples. Boxplot quantification of these targets (**B**) represent interquartile range with median, with whiskers for minimum and maximum non-outlier values. Individual points for each subject are log_2_ transformed and median centered protein intensity values. (PDF 511 kb)

Supplementary Figure 4. Quantification (**A**) and representative blot (**B**) of 4-Hydroxynonenal (4HNE), a marker of lipid peroxidation assessed in skeletal muscle biopsy samples collected from highly-trained endurance athletes following a 4-weeks supplementation with either Urolithin A (UA; n=11) or Placebo (PL; n=9). Resting serum samples were assessed for inflammatory cytokine concentrations (**C**) at baseline (Pre) and following the completion of the training camp (Post, PL; n=20, UA; n=22). Data are means±SEM. (PDF 77 kb)

## Declarations

### Data availability

The authors declare that the data supporting the findings of this study are available within the paper and its Supplementary Information files. All raw data are available from the corresponding authors upon reasonable request.
