# Supplementary figures and images for: Evaluating the Impact of Urolithin A Supplementation on Running Performance, Recovery, and Mitochondrial Biomarkers in Highly Trained Male Distance Runners
Source: Sports Med. 2025 Aug 21;55(12):3183–200. doi: 10.1007/s40279-025-02292-5 (PMC12628386; doi:10.1007/s40279-025-02292-5)

Supplementary Figure 1: CONSORT Diagram

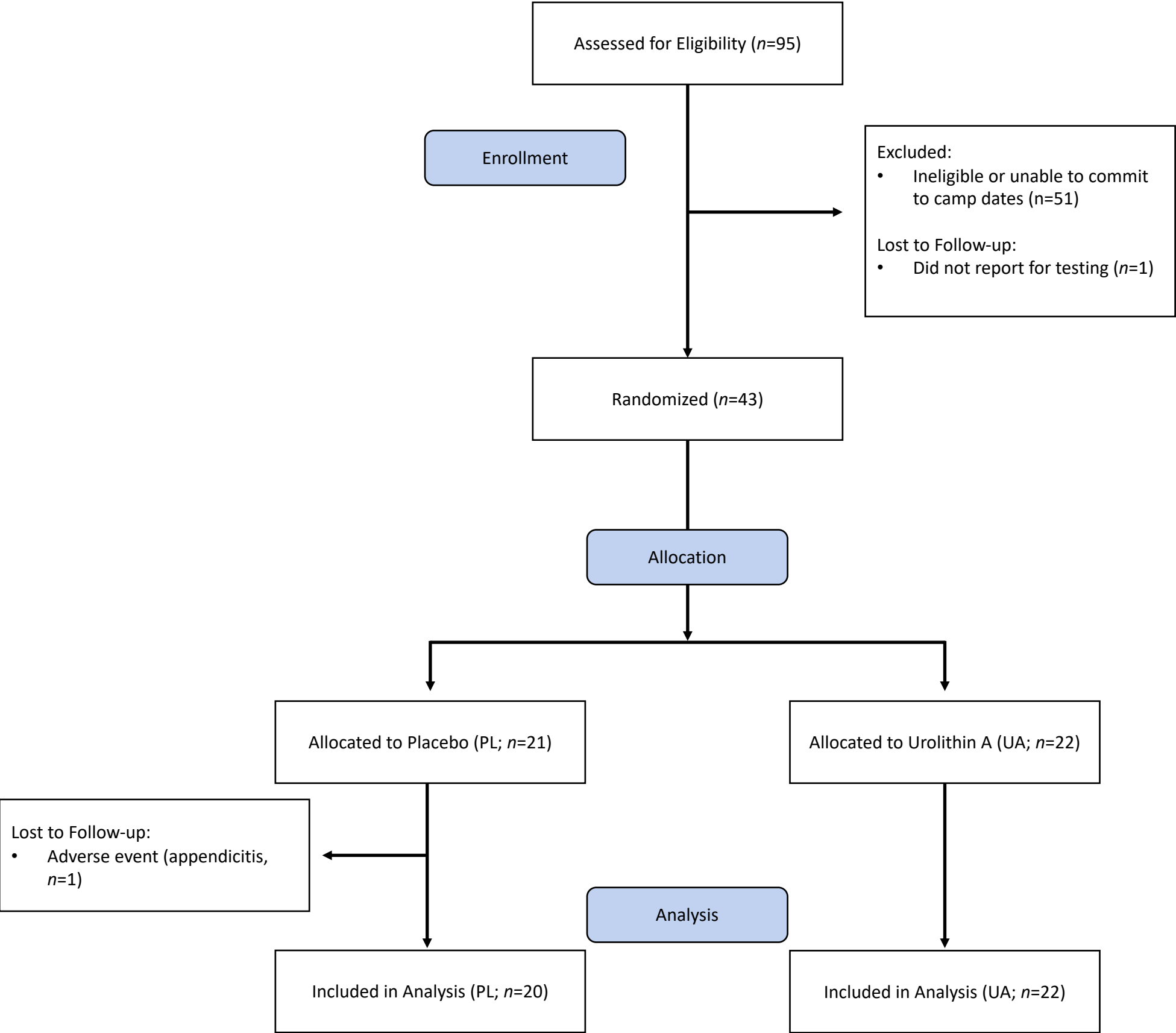

Supplement: Supplementary file 1 — Supplementary Figure 1. CONSORT Diagram (PDF 35 kb) [file 40279_2025_2292_MOESM1_ESM.pdf]

Supplementary Figure 2– PK Analysis

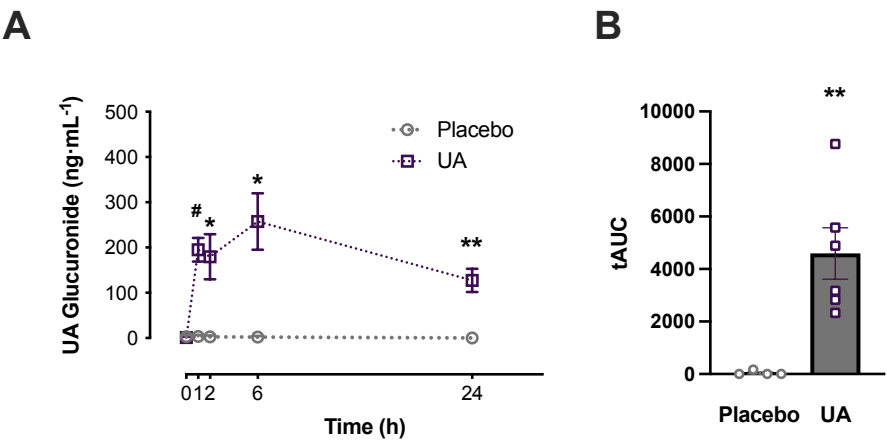

Supplement: Supplementary file 2 — Supplementary Figure 2. Pharmacokinetics of the main urolithin A metabolite UA-glucuronide (panel A) was analyzed in a subset of participants (n=6 UA, n=4 PL) using dried blot spots (DBS) collected on blood collection cards. Fingertip capillary blood samples were collected prior to consuming the first dose of either UA or PL (0 h), and 24 h post-ingestion, with 24 h total area under the curve (tAUC) calculated (panel B). Data are means±SEM, **p<0.01. (PDF 42 kb) [file 40279_2025_2292_MOESM2_ESM.pdf]

Supplementary Figure 3 – Outlier Proteins

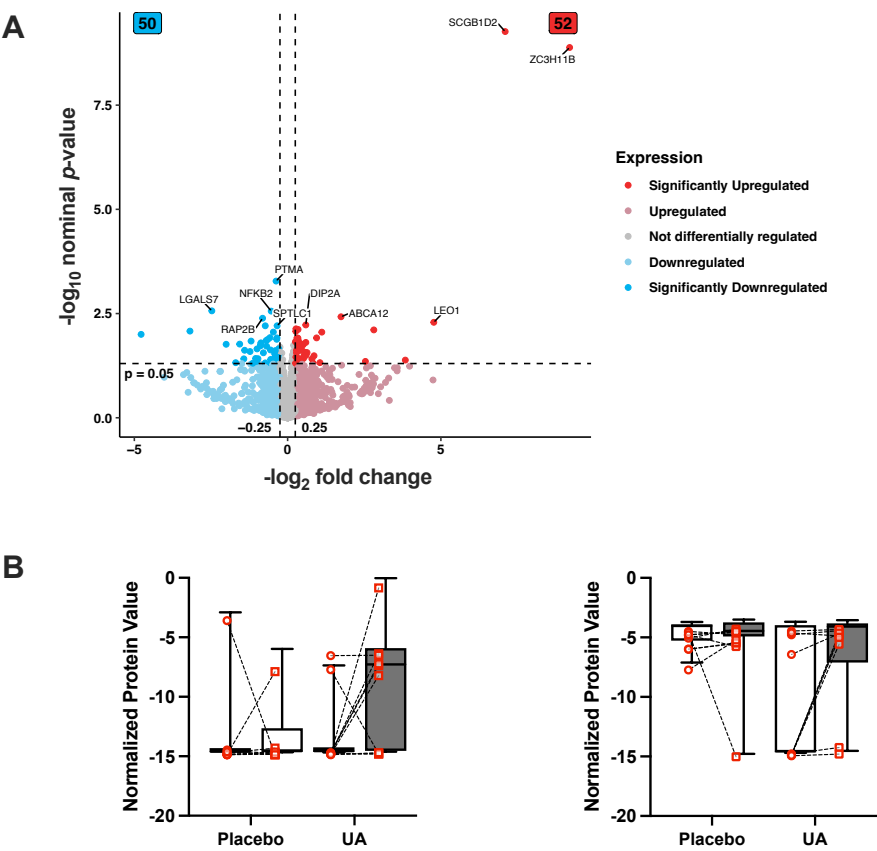

Supplement: Supplementary file 3 — Supplementary Figure 3. Volcano plot (A) showing the median log2 fold change (x-axis) plotted against the –log10 nominal p-value (y-axis) including outliers (SCGB1D2, ZH3H11B). Proteins with an absolute log2 fold change greater than 0.25 and a nominal p-value less than 0.05 were considered significantly regulated. The log2 fold change for ZC3H11B and SCGB1D2 was 9.2 and 7.1, respectively despite sparse expression across samples. Boxplot quantification of these targets (B) represent interquartile range with median, with whiskers for minimum and maximum non-outlier values. Individual points for each subject are log2 transformed and median centered protein intensity values. (PDF 511 kb) [file 40279_2025_2292_MOESM3_ESM.pdf]

Supplementary Figure 4– Inflammation, Lipid Peroxidation

A

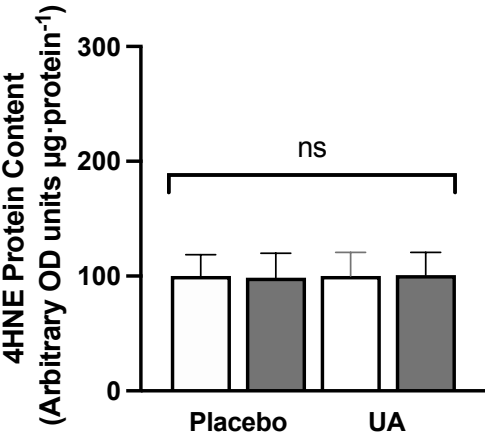

B

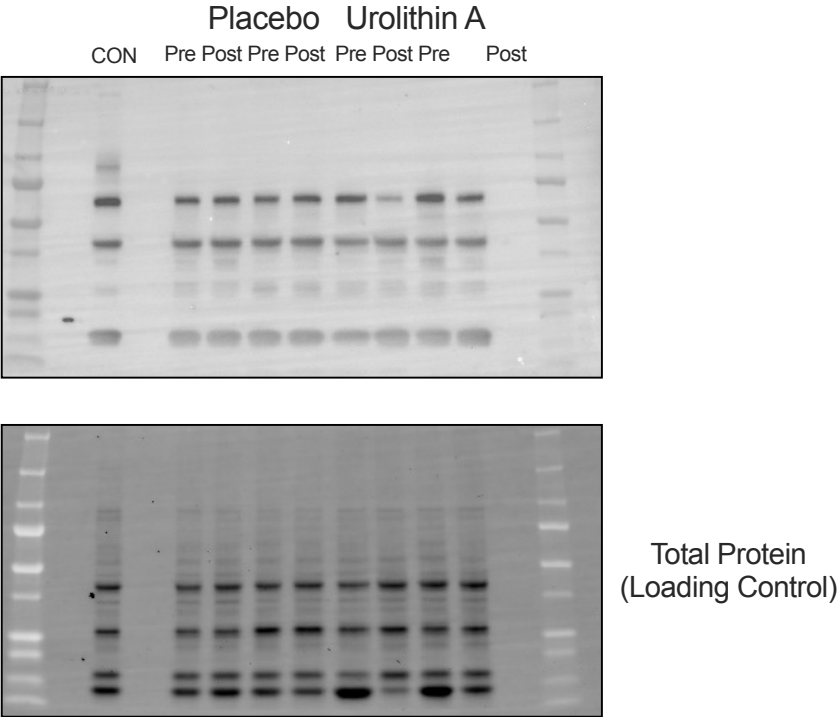

C

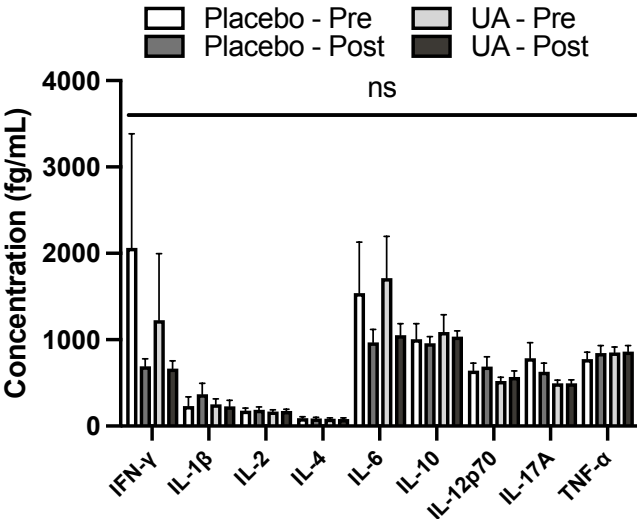

Supplement: Supplementary file 4 — Supplementary Figure 4. Quantification (A) and representative blot (B) of 4-Hydroxynonenal (4HNE), a marker of lipid peroxidation assessed in skeletal muscle biopsy samples collected from highly-trained endurance athletes following a 4-weeks supplementation with either Urolithin A (UA; n=11) or Placebo (PL; n=9). Resting serum samples were assessed for inflammatory cytokine concentrations (C) at baseline (Pre) and following the completion of the training camp (Post, PL; n=20, UA; n=22). Data are means±SEM. (PDF 77 kb) [file 40279_2025_2292_MOESM4_ESM.pdf]
